# Size-Dependent Microwave Heating and Catalytic Activity of Fine Iron Particles in the Deep Dehydrogenation of Hexadecane

**DOI:** 10.1021/acs.chemmater.2c00630

**Published:** 2022-05-13

**Authors:** Xiangyu Jie, Roujia Chen, Tara Biddle, Daniel R. Slocombe, Jonathan Robin Dilworth, Tiancun Xiao, Peter P. Edwards

**Affiliations:** † Inorganic Chemistry Laboratory, Department of Chemistry, University of Oxford, South Parks Road, Oxford OX1 3QR, United Kingdom; ‡ School of Engineering, Cardiff University, Queen’s Buildings, The Parade, Cardiff CF24 3AA, United Kingdom

## Abstract

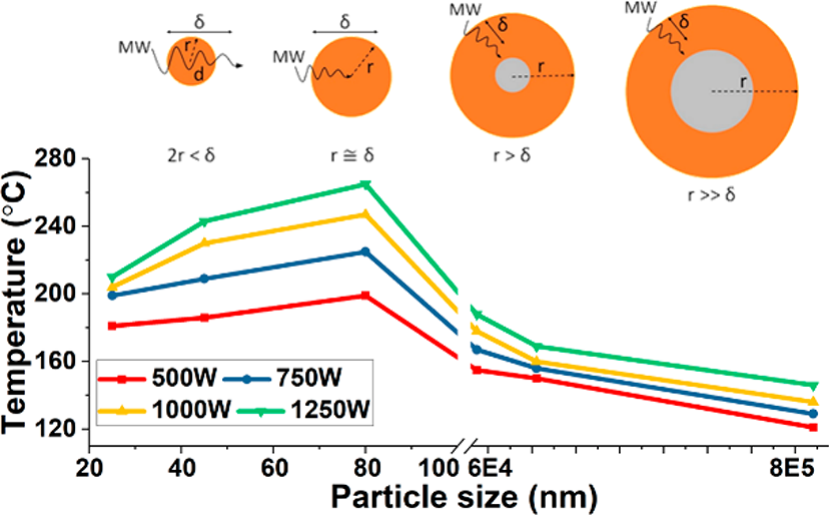

Knowledge of the electromagnetic microwave radiation–solid matter interaction and ensuing mechanisms at active catalytic sites will enable a deeper understanding of microwave-initiated chemical interactions and processes, and will lead to further optimization of this class of heterogeneous catalysis. Here, we study the fundamental mechanism of the interaction between microwave radiation and solid Fe catalysts and the deep dehydrogenation of a model hydrocarbon, hexadecane. We find that the size-dependent electronic transition of particulate Fe metal from a microwave “reflector” to a microwave “absorber” lies at the heart of efficient metal catalysis in these heterogeneous processes. In this regard, the optimal particle size of a Fe metal catalyst for highly effective microwave-initiated dehydrogenation reactions is approximately 80–120 nm, and the catalytic performance is strongly dependent on the ratio of the mean radius of Fe particles to the microwave skin depth (*r*/δ) at the operating frequency. Importantly, the particle size of selected Fe catalysts will ultimately affect the basic heating properties of the catalysts and decisively influence their catalytic performance under microwave initiation. In addition, we have found that when two or more materials—present as a mechanical mixture—are simultaneously exposed to microwave irradiation, each constituent material will respond to the microwaves independently. Thus, the interaction between the two materials has been found to have synergistic effects, subsequently contributing to heating and improving the overall catalytic performance.

## Prologue

John B. Goodenough’s seminal contributions span not only his pioneering work with colleagues at Oxford on lithium-ion batteries, but also his profound influence on our current understanding of the behavior of localized versus delocalized electrons in solids, particularly in the varied roles of d electrons and the associated phenomenon of the metal-to-insulator transition (MIT). There are countless examples in macroscopic systems and materials whereby relatively small changes in thermodynamic parameters (composition, temperature, and pressure) and the resulting electrical conductivity can change by many orders of magnitude as a system transforms from a metallic to a nonmetallic regime across the MIT. Of course, one classic example is the case of VO_2_ where John Goodenough established the fundamental basis of our modern-day description of the scientifically—and technologically—important MIT in that material.

We highlight here also the intriguing possibility of a metal-to-insulator transition driven solely by the physical dimensions of a (nominally) metallic particle. Thus, the inevitable consequence of the successive fragmentation of a metallic particle must be the ultimate cessation of conducting behavior within a mesoscopic or microscopic particle of a critical size. This is the size-induced metal–insulator transition (SIMIT). It is important to note that such an electronic phase transition is driven purely by the physical dimensions of an individual particle. Here, we illustrate that small particles of Fe and Ni catalysts with dimensions characteristic of the SIMIT are efficacious at harvesting microwave energy and converting it into heat. This phenomenon has the desirable effect of maximizing microwave-initiated local heat generation at the active site and significantly enhancing the particle temperature and, with that, the catalytic activity of particles close to the SIMIT. We believe that this phenomenon beautifully highlights John Goodenough’s pioneering approach to the science and technology of materials where several disciplines—here, catalytic chemistry, condensed matter physics, and microwave science and engineering—are intimately related and naturally united in modern multidisciplinary research.

## 1 Introduction

Microwave heating has many advantages over conventional heating, centering on noncontact, selective and rapid heating, and quick start-up and shutdown. The incoming microwaves can be transported from the source through a transparent, quartz, or hollow nonmagnetic metal tube to the sample.^1,2^ However, microwave heating effects on solid state compounds and mixtures are highly complex and are still not fully understood. The ability of microwaves to directly transfer energy and activate specific bond activation via a catalyst, while not significantly heating a surrounding substrate (e.g., hydrocarbons), can be used to alter the thermodynamics of chemical reactions and processes in much the same way as we have traditionally used temperature and pressure and thus can affect catalytic activity and selectivity.

Microwave dielectric heating requires candidate dielectric materials to convert incoming electromagnetic energy to heat thus allowing catalysts to drive reactions under microwave initiation. Several different mechanisms in the interaction, for example, between the electric field of the electromagnetic radiation and materials, can initiate the resulting heating of microwave-responsive molecules and materials.^2−4^ These can include rotational polarization of dipolar molecules, such as water, charge transport, and interfacial polarization in solids known as Maxwell–Wagner polarization.^2−4^

As a result of the many advantages of microwave-initiated catalysis over conventional thermal processes, such as enhanced energy efficiency, higher heating rates, higher reaction rates, and better product selectivity and yield, microwave technology has recently been applied to the important process of CO_2_-free hydrogen production from hydrocarbons.^5^ As the incoming microwave radiation interacts selectively with only the catalytic particles while the hydrocarbon substrates typically remain much colder due to their lack of absorption of electromagnetic radiation, many competing side reactions, including the self-decomposition of hydrocarbons, are significantly reduced. This fundamentally different interaction between microwave radiation and materials will naturally create a temperature gradient between the active catalyst and the substrate or support, and this gradient will enhance the molecular diffusion from, and heat transfer within, the sample.^1,6^ However, it appears to be of great importance to select a catalyst for microwave-initiated reactions which meets specific requirements. These are in addition to the necessary high activity, selectivity, and stability required of operating catalysts, and such a catalyst must be a good microwave susceptor and be able to retain its structure and properties under intense microwave irradiation.^7,8^ In general, metal- and carbon-based catalysts have been widely used.

Ménéndez et al. reported on microwave heating processes involving carbon materials,^9^ which show an excellent ability to absorb microwaves, and because of this carbon materials are commonly used as microwave susceptors which heat other materials indirectly, or they themselves directly act as a catalyst in different heterogeneous reactions. A variety of carbon materials, such as activated carbons, metallurgical cokes, chars, or anthracite, were used for several microwave-enhanced heterogeneous catalytic reactions.^9^ Domínguez et al. investigated the microwave-assisted decomposition of methane on activated carbon, which produced a higher selectivity of H_2_ and higher conversions compared to electrical or conventional heating.^10^ The improved catalytic performance was attributed to the formation of hot spots inside the catalyst bed which favor CH_4_ decomposition. Jie et al.^11^ studied various carbon materials and residues with different geometries and structures and examined their initial interaction and catalytic performance when used in dehydrogenation reactions under microwave irradiation. However, even though all the tested carbon materials displayed excellent microwave absorption properties, only a few types of carbonaceous materials, such as activated carbons and graphene nanoplatelets, appeared to be catalytically active for the microwave-initiated dehydrogenation of hexadecane.

In studies on metal catalysts in microwave-initiated heterogeneous catalysis, Suttisawat and Horikoshi et al. showed efficient hydrogen production from the dehydrogenation of various organic hydrides, including methylcyclohexane, tetralin, and decalin, over carbon-supported Pt and Pd catalysts.^1,12−14^ The tetralin conversion was significantly enhanced from 31% in conventional thermal heating to 56% under microwave irradiation.^13,14^ This enhancement was explained by the highly effective heating ability of Pt/carbon particles which created a large-temperature gradient between the catalyst and the tetralin solution under microwave conditions. Horikoshi et al.^12^ achieved over 94% conversion of methylcyclohexane using a Pd/AC catalyst at 340 °C in 2 min.

Research by Gonzalez-Cortes et al.^15^ proposed using long-chain alkanes, such as paraffin wax (>C_20_ alkanes), as a benign hydrogen storage material; it was demonstrated that the rapid liberation of hydrogen could be achieved via microwave-initiated catalysis of the paraffin wax. A ruthenium (Ru/AC) catalyst on an activated carbon support was prepared and used for hydrogen production using paraffin wax (C_26_H_54_) decomposition under microwave irradiation. A hydrogen concentration of 60–80 mol % was obtained in the exit gas stream over the Ru/AC catalyst. In contrast, only approximately 40 mol % of hydrogen was produced when only an activated carbon catalyst, without the ruthenium metal, was used. The authors attributed this to the large availability of surfaces of the activated ruthenium metal nanoparticles.^15^ Jie et al.^16−18^ used iron, nickel, and alloys of these metals supported on silicon carbide (SiC) as catalysts to dehydrogenate fossil hydrocarbons to produce high-purity hydrogen under microwave irradiation. A very high selectivity for H_2_ production, 98% of all evolved gases, was achieved; this was much higher than using conventional thermal processes. In addition, the authors noted that the catalytic performance under microwave irradiation was varied by using different metals, support materials, and metal loadings.

The difference between carbon-based and metal-based catalytic systems for microwave-initiated catalysis has also been studied by Tanner et al., who reported on the microwave-assisted catalytic reactions of a variety of liquid hydrocarbons (straight-chain, branched, aromatic, and functionalized) using millimeter-sized chars. A vigorous emission of sparks was generated over the chars while under microwave irradiation and resulted in molecular hydrogen, gaseous hydrocarbons, and a range of liquid olefins.^19,20^ Of these products there was mainly hydrogen, ethylene, and α-olefins present in the liquid products. The content of α-olefins was more than an order of magnitude greater than the content of other products, mostly alkanes. They associated the high α-olefin selectivity to the spark discharge that arose on the carbon surface upon microwave irradiation; this caused C–C or C–H bond cleavage to form alkyl radicals and hydrogen atoms in a short reaction time period.^20^ However, Udalov et al. argued that the exclusive selectivity of hexadecane pyrolysis to hydrogen, ethylene, and α-olefins might also be a consequence of the catalytic action of a variety of naturally occurring metals contained within the carbon. This was supported by the proposal that metallic catalysts under microwave irradiation involve hot spots which promote the thermal reactions of methane, which leads to the formation of gas-phase methyl radicals and hydrogen atoms.^21^ Therefore, an expanding range of catalysts, including Fe_2_O_3,_ magnetic microspheres (coal combustion ash, 92.5% mass % Fe_2_O_3_ with major impurities: 4.9% CaO, 1.3% MgO, 1.3% SiO_2_), glass fiber supported palladium catalysts, metal-ceramic Al_2_O_3_/Al catalysts, ZSM-12 zeolite catalysts, and 70% ZSM-5/Al_2_O_3_ (0.2–1.0 mm fraction), were investigated for microwave-assisted hexadecane pyrolysis. As a result, methane, ethylene, and propylene were found to predominate in the gaseous reaction products, and similarly, α-olefins were mostly observed in the liquid products of the hexadecane pyrolysis when using magnetic microspheres and metal-ceramics.^21^ These results again indicate the great importance of choosing an appropriate catalyst for microwave-initiated catalysis.

With a better understanding of an active catalyst’s interactions with microwave radiation, we can more effectively and specifically optimize its catalytic performance. Here, we studied the important effect of the particle size of metal catalysts and the initial interaction of two or more materials/substances under microwaves; in all cases, the temperature profile and microwave energy absorption changes were monitored. The corresponding catalytic performance of using different sized Fe particles, a mixture of two or more materials, and different supporting materials for the metal catalysts were also investigated, and the results are presented here.

## 2 Materials and Methods

### 2.1 Materials

The different sized iron particle powders, including 25 nm, 35–45 nm, 60–80 nm, 200 mesh (74 μm), 70 mesh (210 μm), and 20 mesh (841 μm) iron particles, and activated carbon (AC) were bought from Sigma-Aldrich and used as purchased without further treatment. The mixture sample of iron and activated carbon was prepared by physically mixing a certain amount of iron particles and activated carbon, and the mixture was then vigorously blended using a pestle and mortar.

The AC and silicon carbide (SiC) supported metal catalysts were prepared using the so-called incipient wetness impregnation method. Iron nitrate, Fe(NO_3_)_3_**·**9H_2_O (iron(III) nitrate nonahydrate, Sigma-Aldrich), and nickel nitrate, Ni(NO_3_)_2_**·**6H_2_O (nickel(II) nitrate hexahydrate, Sigma-Aldrich), were used as metal precursors. AC (activated carbon, Sigma-Aldrich) and SiC (silicon carbide, Fisher Scientific), of the SiC–6H structure, were used as supporting materials. In the preparation, the supporting material was mixed with an aqueous solution of metal nitrate, the concentration of which was calculated to produce the desired metal loading. The mixture was stirred at 150 °C for 3 h on a magnetic hot plate until it became a slurry. The slurry was then moved into a drying oven and left overnight. The resulting solid mixtures were calcined in a furnace at 350 °C for 3 h. Finally, the active catalysts were obtained by a reduction process in 10% H_2_/argon gases at 800 °C for 6 h.^16,17^

### 2.2 Material Microwave Response Test

The temperature profile and delivered MW power of materials under microwave irradiation were recorded by exposing the samples to different microwave power. The experimental setup was reported previously and consists of a microwave generation system, a purpose-built microwave cavity, and a control system.^15,16^ The sample materials were loaded into a quartz tube with an inner diameter of 6 mm and a 9 mm outer diameter. The typical volume of samples loaded was approximately 1.13 cm^3^ with a height of 3–4 cm; this enables the sample bed to be fully exposed to the uniform axially polarized (TM_010_) electric fields in the purpose-built aluminum cavity. The samples were first purged using an Ar flow at a rate of 1.67 mL·s^–1^ for a period of 15 min. The samples were then irradiated with microwave irradiation at different input powers for 10 min. The sample temperature was recorded using an infrared (IR) pyrometer, which was also used to control the power of the generator. A Labview software program was linked to control the microwave system, and the temperature (*T*), input power (W), and dissipated power (W) versus time (*t*) profiles were recorded with this program.

### 2.3 Material Catalytic Activity Test

The material catalytic activity tests were carried out using the same experimental setup and quartz tube as discussed in the microwave response test. In addition to the sample preparation, a certain amount of hexadecane (approximately 30 wt % of catalyst samples, 0.3–0.5 mL) was mixed/injected into the tube and left for 5–10 min until the liquid hexadecane was well dispersed into the catalyst bed. The filled tube was then placed axially in the center of the TM_010_ microwave cavity to minimize depolarization effects under microwave irradiation. Before the microwave generator was started, the samples were purged with an Ar flow at a 1.67 mL·s^–1^ rate for a period of 15 min. The sample was then irradiated with microwaves for 10 min at an input power of 750 W. The evolved gas products were collected and analyzed using a gas chromatographer (GC, PerkinElmer, Clarus 580 GC). The unreacted “escaped” hydrocarbon feedstocks were recycled, and their composition was confirmed by a gas chromatograph mass spectrometer (GC-MS, SHIMADZU, GCMS-QP2010 SE).

We determined the catalytic performance of a material sample under microwave irradiation as the hydrogen production in terms of the hydrogen concentration in the evolved gas products. The selectivity toward different gaseous products was evaluated in terms of the volume % of the product composition in the exit gases that had been analyzed by GC (eq 1).
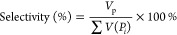
1*V*_p_ is the volume % of one gas product obtained from gas chromatography (GC), and ∑*V*(*P*_*i*_) is the sum of the volume % of all the products in the exit gases.

## 3 Results and Discussion

### 3.1 Temperature Profiles of Fe Catalyst Particles under Microwave Irradiation

#### 3.1.1 Free-Standing Fe Particles

Unlike high dielectric loss factor materials (e.g., water) that can efficiently absorb microwaves, bulk metals are themselves impenetrable to microwaves (beyond their characteristic skin depth), primarily owing to the presence of an itinerant, free-electron cloud which causes broad-band reflection at microwave frequencies. Thus, bulk metals are usually reflectors of microwaves. In 1988, Gleiter et al.^22^ first reported a drastic decrease in electrical conductivity with particle diameter below a few micrometers; in later years, Edwards et al.^23^ proposed that by varying the size of the individual mesoscopic particles, any value of their conductivity can be adjusted between the full extremes of an insulator and a metal below that of the corresponding bulk metal. This is known as the size-induced metal–insulator transition (SIMIT) and forms the fundamental physical basis for the possible transition of metals from a “reflector” of microwave irradiation to an “absorber” in the mesoscopic regime.

**Figure 1:**
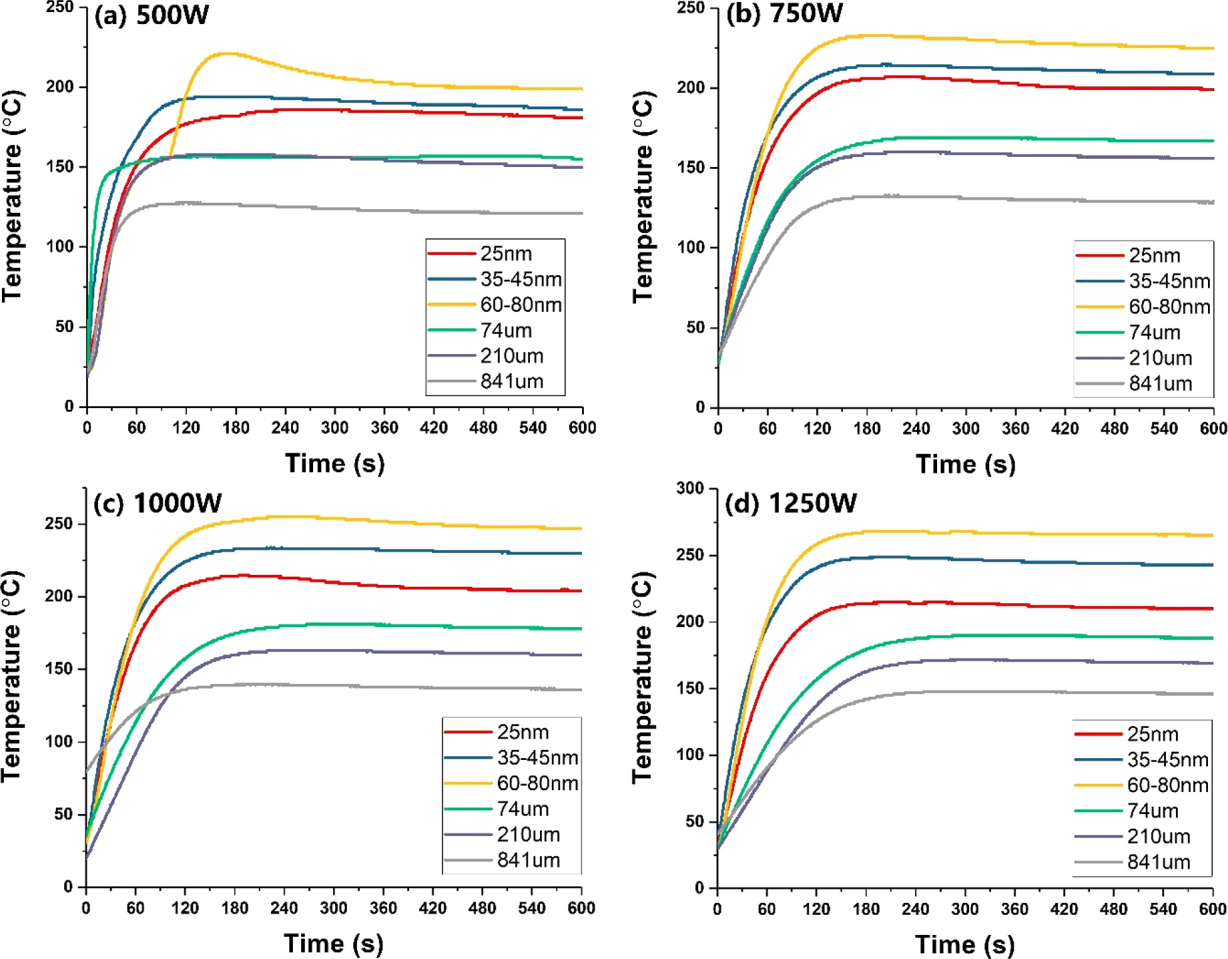
Temperature profiles of Fe particles of various sizes under microwave irradiation with different input powers of (a) 500, (b) 750, (c) 1000, and (d) 1250 W.

In Figure 1, we present the heating profiles of different sized Fe particles when exposed to microwave irradiation at various microwave input powers. For a SIMIT, the successive fragmentation, or division, of metal particles will ultimately inhibit and then reduce completely the conducting behavior of metals. It is this transition from the metallic (macroscopic) through to the insulating (mesoscopic and microscopic) extremes that will ultimately enable/improve the microwave absorption of nominally metallic catalyst particles.^24^

Arising from such a SIMIT, Fe particles on the nanoscale achieved noticeably higher temperatures than those on the meso- or microscale. The largest of the Fe particles, 841 μm, heated to approximately 120–140 °C at various microwave input powers, while the temperature achieved by the 60–80 nm sized Fe particles was always approximately 100 °C higher. However, we note that the temperature of the Fe particles was not a linear function with respect to the particle size (Figure 3c). On the nanoscale (<100 nm), the temperature of the Fe nanoparticles and their size have an almost linear relationship; as the particle size was reduced the temperature also decreased. From the data we can see that the optimal size of Fe particles which presented the most rapid heating profile was 60–80 nm (Figures 1 and 3); the 60–80 nm particles reached the highest temperature at each of the different microwave input powers.

The temperature profiles of the different sized Fe particles reflect the sample’s microwave-absorbing properties because the heat is generated by the conversion of absorbed electromagnetic energy. In the interaction of microwaves and metal particles, the skin depth of the sample material must be carefully considered; the skin depth (δ) describes the distance at which the microwave power decreases to 1/*e* of its power value at the surface.
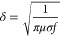
2Here, δ is the skin depth, μ is the permeability of the conductor, σ is the conductivity, and *f* is the frequency. Thus, for any conductor like iron with high permeability and conductivity, the skin depth is very small, usually between the nanoscale and microscale for a GHz frequency.^25,26^

Porch et al.^27^ calculated electromagnetic absorption in small conducting particles within microwave fields for a range of particle sizes and conductivities. It was concluded that the magnetic absorption dominates electrical absorption over a wide range of radii for highly conducting particles with an optimum absorption set by the ratio of the mean particle radius to its skin depth (*r*/δ). This indicates that for metal particles of any conductivity, optimized magnetic absorption, and hence microwave heating by magnetic induction, can be achieved by the simple selection of the mean particle size.

**Figure 2:**
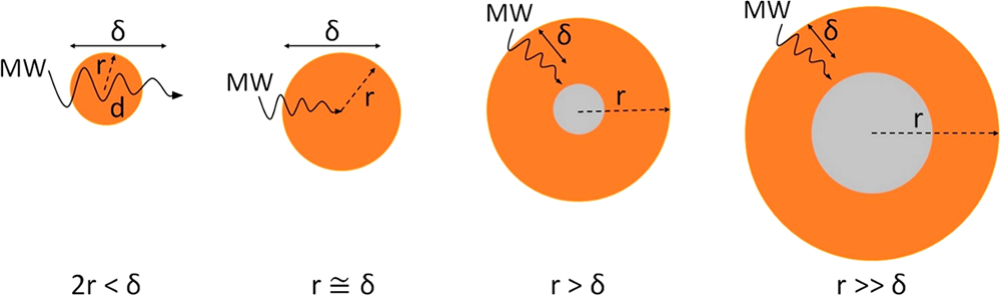
Schematic representation of the microwave penetration in the different sized Fe particles having the same skin depth at 2.45 GHz frequency. “MW” indicates incident microwave power, while *r* and δ are the radius and skin depth of iron particles, respectively.

**Figure 3:**
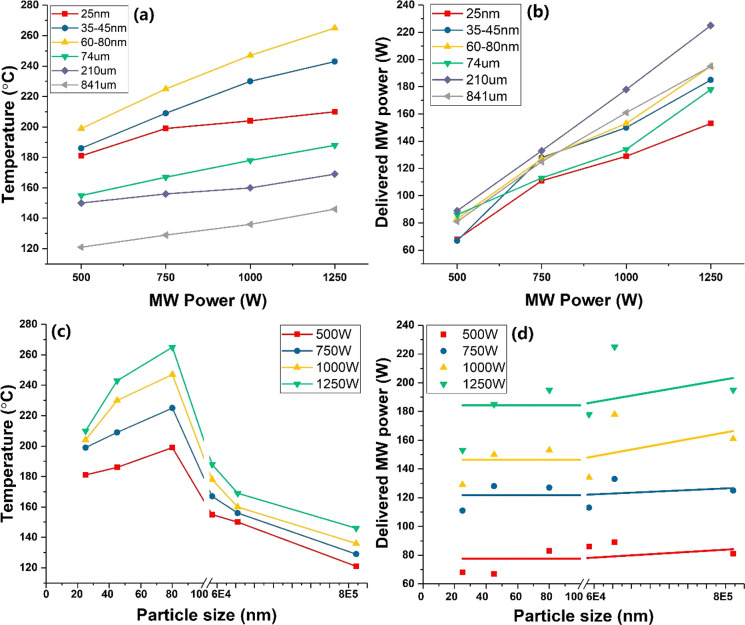
Study on various sized Fe particles exposed to microwave (MW) irradiation, showing (a) the temperature and (b) the delivered MW power of samples recorded at 600 s, in the balanced heat/energy environment, as a function of microwave input powers. (c) The temperature recorded at 600 s as a function of Fe particle size. (d) The delivered MW power of samples at 600 s as a function of Fe particle size.

In the experimental demonstration, we showed that the temperature achieved among different sized Fe particles was 80–60 nm > 45–30 nm > 25 nm > 74 μm > 210 μm > 841 μm (Figures 1 and 3c). At our operating microwave frequency of 2.45 GHz, different sized Fe particles have the same skin depth δ, approximately 41.5 nm.^28^ Thus, the volume of the Fe particle, which absorbs incoming microwave power, is closely related to its particle size and skin depth (Figure 2). When the diameter of a Fe particle is smaller than its skin depth (2*r* < δ), the full volume of the particle will absorb the microwave radiation and generate heat. However, on the nanoscale of the tested Fe samples, smaller particle sizes of Fe produced lower temperatures. This is because as the particle’s size is increased, a greater volume of material will present itself to absorb the microwaves until the radius becomes larger than the skin depth. This again demonstrates the importance of the ratio of the mean particle radius and the skin depth (*r*/δ),^27^ which will affect the heating properties of Fe particles under microwave irradiation. In contrast, on the microscale, the temperature reached by Fe particles decreased as the particle size increased; this is not only because at the macroscopic level the Fe metal particles will also exhibit conducting behavior that reflects microwaves without heating but also because the fraction of the particle’s volume that is available to absorb electromagnetic energy is smaller in large Fe particles and therefore presents poor heating.

Conventional heating is a slow process—particularly for a heterogeneous mix of a metal catalyst particle + a low (thermal conductivity) absorption hydrocarbon (also, in excess, of course)—necessitating the conduction of heat from the exterior surface of the mixture to the interior to ultimately reach the catalyst particle. In contrast, microwave heating is more efficient; it is driven by the direct absorption and transfer of microwave energy within the interior of the heterogeneous mixture. Hence, incoming electromagnetic energy is directly transferred to the catalyst particle without depending on such a heat transfer process. Within this study, the Fe particles were heated within 30–120 s and stabilized at the relevant temperatures. This can be seen from the plateau on the graphs, at which a balanced/equilibrated heat/energy environment was built up (Figure 1). Thus, we recorded the temperature and delivered MW power after 600 s as a representative example of that balanced heat/energy environment.

We note that in this study, “delivered MW power” was taken from the microwave input power *minus* the reflected power to illustrate the evolved change of microwave powers that were delivered to the cavity. In relation to the wave impedance matching, the change of the sample’s permeability, permittivity, physical dimensions, and temperature can affect the actual amount of microwave power delivered to the cavity.

As a general observation, both the temperature of the samples and the delivered MW power in the plateau region of the graphs are almost linearly increased when using a higher microwave input power (Figure 3a and b). Increasing the microwave energy supply will generate more heat at the catalyst particle and thus will result in higher overall temperatures of the samples. The Fe metal particles act as energy converters, which constantly generate heat by the absorption of electromagnetic energy. Of course, this is a dynamic process of equilibration as the “hot” metal particles—as well as undergoing a catalytic dehydrogenation process—will also cede/transfer thermal energy to the surrounding hydrocarbon “lattice”. This illustrates the inherent complexity of attempting to model the entire processes under microwave-initiated catalysis.

In Figure 3c and d, we show the resulting temperature and delivered MW power of Fe particles as a function of their particle size. When the ratio of the mean radius of Fe to its skin depth (*r*/δ) is below 1, on the nanoscale, smaller particles produced less heat; at the Fe particle size of 60–80 nm, where the *r*/δ ratio is approximately 1, the highest temperature among all the tested samples was reached.

In contrast, the micrometer-sized Fe particles (*r*/δ>1) were barely heated under exposure to microwaves because these large particles were not fully penetrated by microwave irradiation. Interestingly, the delivered MW power remained almost constant for all the different sized particles, suggesting that it is independent of Fe particle size. However, the samples containing the smallest Fe particles, of characteristic size 25 nm, reflected the most microwaves. Therefore, for metal particles exposed to microwaves, the optimum particle size for heating is approximately 2–3 times that of the material’s skin depth, and the optimum absorption is determined by the ratio of the mean particle radius to the skin depth (*r*/δ). With this knowledge, particle size can be carefully varied (“tuned”) to achieve more efficient heating.

#### 3.1.2 Physically Mixed Fe Metal Particles and Activated Carbon (AC)

In microwave-initiated heterogeneous catalysis,^9,17,29−31^ microwave irradiation will directly and separately interact with different substances depending upon their unique dielectric properties. This fundamental nature of the interaction of microwaves with substances gives possible synergistic effects of heating when a mixture of two or more materials is exposed to the microwaves. Particularly, during the microwave heating of a solid substance, interfacial polarization occurs at the surface of the microwave-absorbing particles; this will therefore cause a major local increase in the electric field strength and hence dramatic differences in heating rates between the different constituents at their interface.^18,32^ To elaborate on this, we selected two kinds of Fe particles with different particle sizes of 60–80 nm and 841 μm, which were physically mixed with activated carbon (AC) in a weight ratio of 1:1. The mixture samples were then tested under different microwave power inputs.

**Figure 4:**
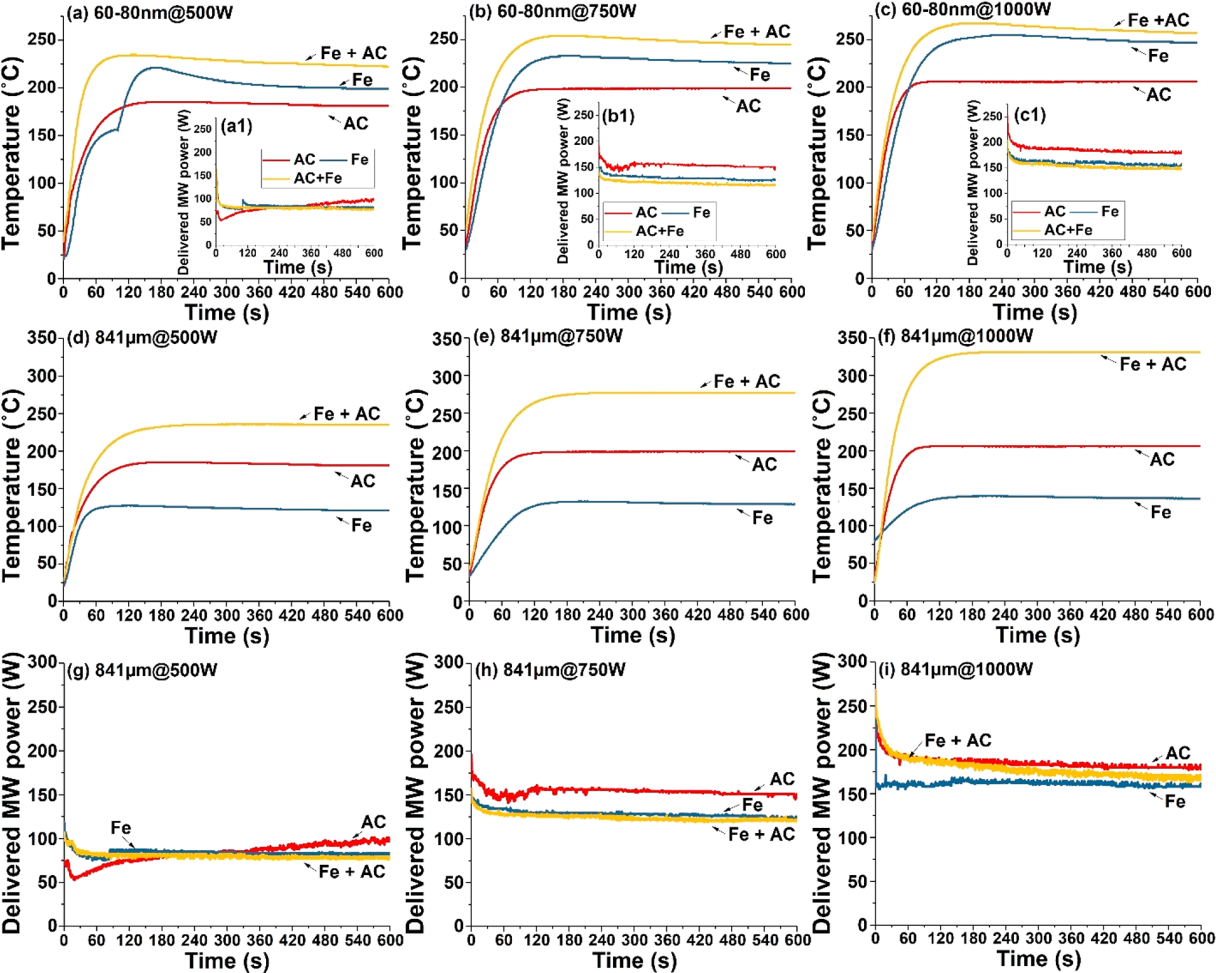
Temperature profiles of activated carbon (AC), 60–80 nm Fe particles, and their mixture (Fe + AC) under microwave with different input powers: (a) 500, (b) 750, and (c) 1000 W. Inset figures (a1, b1, and c1) correspond to delivered MW power. (d−f) Temperature profiles of activated carbon (AC), 841 μm Fe particles, and their mixture (Fe + AC) under 500, 750, and 1000 W microwave power inputs and (g–i) corresponding to delivered MW power. The mixing weight ratio of Fe and AC was 1:1.

In Figure 4, we show the temperature profiles and delivered MW power of Fe and carbon mixtures exposed to microwaves. As a general—but important—observation, the temperature of the mixture (Fe + AC) was higher than that of the pure, constituent Fe particles and AC powder. The temperature gap between the mixture and its constituent materials was more obvious when Fe had a larger particle size (on the microscale).

**Figure 5:**
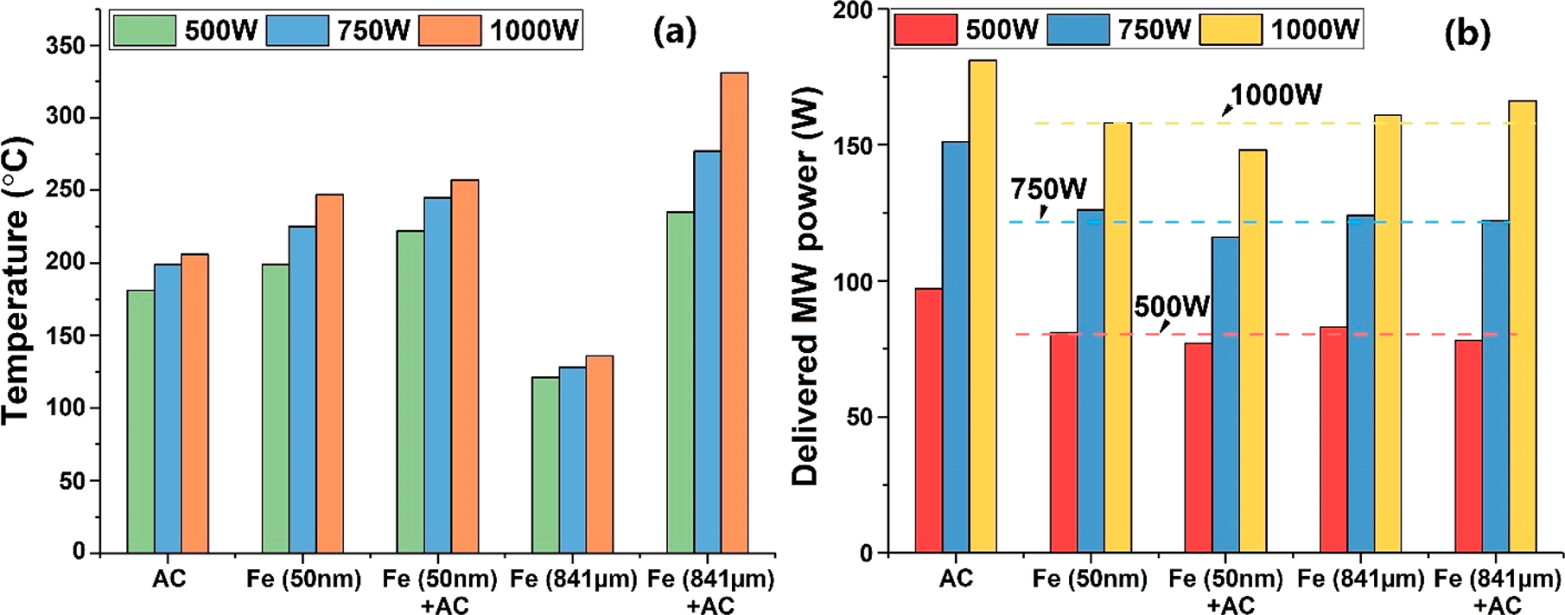
Balanced heat/energy stage (a) temperature and (b) delivered MW power taken at 600 s of representative physically mixed samples.

Importantly, the observed temperature of the 841 μm Fe and AC mixture was approximately 100–200 °C higher than that of their constituent (separated) samples. However, interestingly, there were few differences found in the delivered microwave power: particularly, the delivered microwave power of the mixture, which is nearly the same as that of the pure Fe particles. To make a comparison of this discovery between the physically mixed materials and the microwave input power, we then collected the point data at 600 s, which we take as the balanced heat/energy stage, and these data are given in Figure 5.

For particles at the nanoscale, compared to both the Fe particles and the AC powder alone, the temperature was mildly increased when both materials were mixed (Figure 5a). In contrast, micrometer Fe particles (841 μm) heated poorly because microwave irradiation has been screened by the electron cloud of the large Fe particle, but the mixture with AC significantly improved its heating effect. We ascribe this to the synergistic enhancement from the surface interfacial polarization between Fe and AC particles. Our previous simulations^18^ presented that the electric field strength and the heating rate at the interface of the two substances increased significantly due to interfacial polarization. High localized electric fields and increased heating rate will occur between the particle surfaces.^27^

Moreover, despite the increased microwave input power supply, the delivered MW powers of two Fe particles sizes, one 60–80 nm and the other 841 μm, and their mixture with AC were nearly the same (Figure 5b). This suggests that for the same metal materials, the change of particle size (over its skin depth at operating frequency) and mechanical mixing with another material (herein AC) cause a negligible change in the impedance match presented by the microwave cavity for Fe and Fe + AC samples. We attribute this to the dependency on the intrinsic characteristic skin depth of metals at the operating frequencies; here, at 2.45 GHz, the skin depth of iron is approximately 41.5 nm.

Thus, where the particle size of both Fe particles exceeds the value of its characteristic skin depth, they primarily reflect the microwaves. One can imagine that not only will a large metal particle have increased electrical conductivity which increases the reflectivity to microwaves, but also the interior core of the metal particle will remain cold; this is a result of the limited microwave penetration and energy absorption which cannot heat up the entire particle (Figure 2). Importantly, when the physical dimensions of the particles are less than their skin depth at the operating frequency, the microwaves will penetrate throughout the entire particle. If the particle size is increased, optimal heat generation will occur as the larger volume of particle material absorbs more of the microwave energy. However, a further increase in the particle size leads to the onset of the skin effect which excludes the microwaves from the particle core and again leads to lower temperatures (Figure 3c).

Despite almost the same delivered MW powers among the Fe particles and the mixture of Fe and AC, higher temperatures were observed for the mechanical mixture samples. This indicates that the synergistic effect from surface interfacial polarization of two constituent substances, under microwave irradiation, will generate heat. Furthermore, nonequilibrium plasma could potentially be generated at the interface of the two substances, initiated by the microwave irradiation;^18,33^ the combination of plasma and solid metal particles under microwave irradiation has been found to have synergistic effects and enhance the average electric field strength.^33^ Surface absorption within a plasma volume can also make a significant contribution to heating.^18^ Unfortunately, we note that it is difficult with current characterization techniques to distinguish the difference between the synergistic effect by surface interfacial polarization and the plasma effect. Further studies that take into account the interfacial chemistry and nonequilibrium kinetics under a microwave electric field need to be undertaken, and we are currently addressing this issue.

#### 3.1.3 Fe and Ni Particles Supported on AC and SiC

To further study the combination of two solid substances at a close physical contact/proximity, under microwave initiation, we prepared activated carbon (AC) and silicon carbide (SiC) supported metal catalysts. Iron and nickel metal particles were loaded through an incipient wetness impregnation method (see Section 2.1 for details) The samples were then tested under microwave irradiation at a system input power of 750 W.

**Figure 6:**
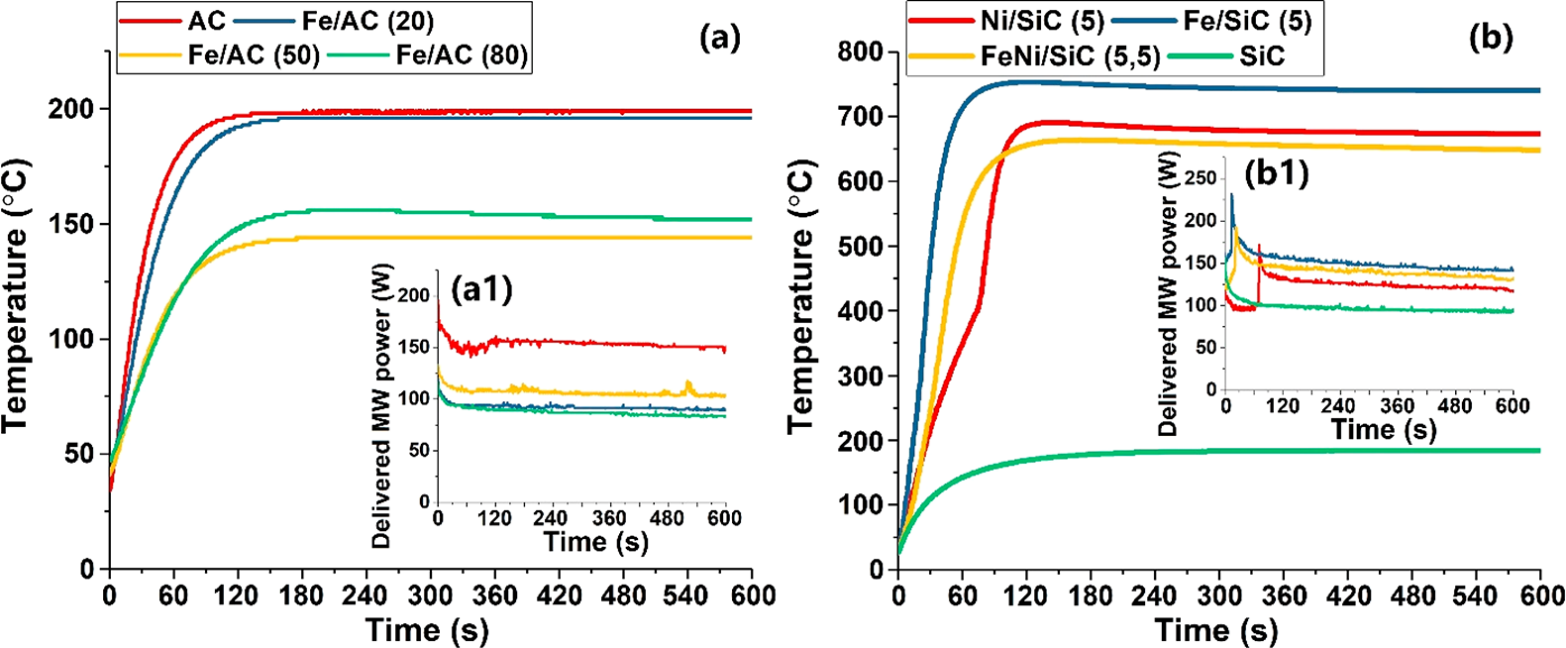
(a) Temperature profiles of activated carbon (AC) supported Fe particles, with different Fe loadings. (b) Temperature profiles of silicon carbide (SiC) supported 5 wt % Fe, Ni, and Fe–Ni alloy particles. Inset figures (a1 and b1) correspond to the delivered MW power. Microwave input power was 750 W. Numbers in parentheses indicate the metal loadings in wt %.

Unlike the mechanical mixture of Fe particles and AC powders, AC-supported Fe particles showed poor heating under microwaves. At the lower Fe loading of 20 wt %, the resulting temperature was similar to pure AC powders, and the temperature decreased to about 150 °C when Fe loadings were increased to over 50 wt %. We ascribe this to the resulting large Fe particles in the high Fe loading samples. Moreover, we note that the delivered MW powers (Figure 6a1) were decreased when Fe metal was loaded; the sample will not heat well because of the less efficient impedance match, and the increasing Fe loading leads to poor coupling.

In contrast, the SiC-supported Fe and Ni particles illustrate the opposite scenario, an incredibly rapid rise in temperature. Importantly, we note that this spectacular temperature rise occurs *only* for metal particles on the SiC support; no such effect is seen for the SiC support by itself. The characteristic physical dimensions of loaded Fe and Ni particles are about 100–150 nm at 5 wt % metal loading, and the size of SiC is about 20–40 μm.^5,16^ The temperature gap observed between the SiC powder and SiC-supported metal (Fe and Ni) particles and the Fe particles under microwave irradiation is over 500 °C. We believe the proximal contact of iron particles and SiC support causes synergistic surface effects leading the supported metal catalysts to exhibit heating properties above that of the independent constituent material. Moreover, the delivered MW power increased significantly (Figure 6b1) at the start of the microwave exposure which indicates the proper combination of Fe nanoparticles and SiC support leading to wave impedance matching; hence, more microwave energy was delivered.

**Figure 7:**
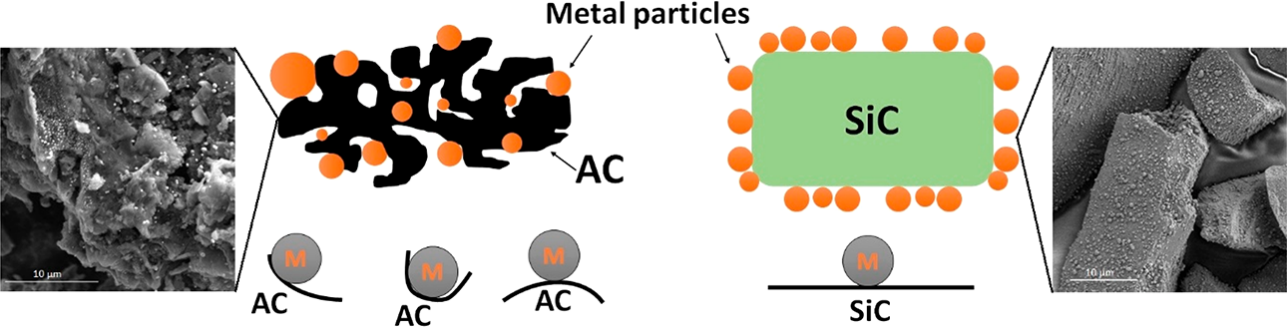
Diagram showing the possible nature of the interaction of metal particles and the different supporting materials AC and SiC.

The use of different supporting materials, AC and SiC in this study, suggests that the material’s structure and shape impact the interaction with incoming microwave irradiation. Han et al.^34^ studied the shape effect of Fe particles on their electromagnetic properties and concluded that the shape of particles affects both permittivity and permeability values and therefore, changes microwave absorption performances. Comparing the supports of AC and SiC, AC is a porous material with a very high surface area, but SiC is a high mechanical strength nonporous support with low surface area.^11,16^ When metal is loaded via an incipient wetness impregnation method, metal particles will anchor into the pores or unevenly attach to the rough surface of AC. The SiC has a smooth and flat surface where metal particles are in a “touchdown” arrangement (Figure 7). Thus, the structural differences of constituent materials could vary the interfacial properties between the supports and metal particles and therefore, contribute to the differences in microwave absorption and heating.^34−37^

### 3.2 Catalytic Performance of Free-Standing, Mechanically Mixed, and Supported Catalyst Particles

The corresponding catalytic activities of these three different types of iron-based metal catalysts, namely, free-standing, mechanically mixed, and supported catalyst particles, were tested in the representative microwave-initiated dehydrogenation of a model hydrocarbon (here, hexadecane). With the burgeoning interest in clean hydrogen production, ideally with no CO_2_ emissions, our previous studies^5,16^ on microwave-initiated dehydrogenation of hydrocarbons crucially showed that high-purity hydrogen can be rapidly liberated from liquid hydrocarbons over iron-based metal catalysts. In the absence of microwave initiation in the catalytic process, the hydrogen yield was significantly decreased with higher concentrations of light alkanes produced; this indicates that thermal cracking was dominant in the conventional heating process.

Two advances in utilizing microwaves for CO_2_-free hydrogen production from hydrocarbons can be identified. The first is the efficient energy transfer from the incoming microwaves to the catalyst particles enabling rapid heating on the particle surface, where the initiation of the catalytic reaction also occurs. The second important advance is the apparent, effective limitation or inhibition of side reactions of self-decomposition of hydrocarbons which would occur at higher temperatures. Under microwave-initiated conditions, hydrocarbons are essentially transparent to microwave irradiation, and the “bath” will initially remain cold before contacting the hot catalyst particles. As a preferred mechanism of the microwave-initiated process, catalytic scission of chemical C–H bonds in hydrocarbons^18^ significantly improves the selectivity to H_2_ and hence narrows the chemical product distribution.

**Figure 8:**
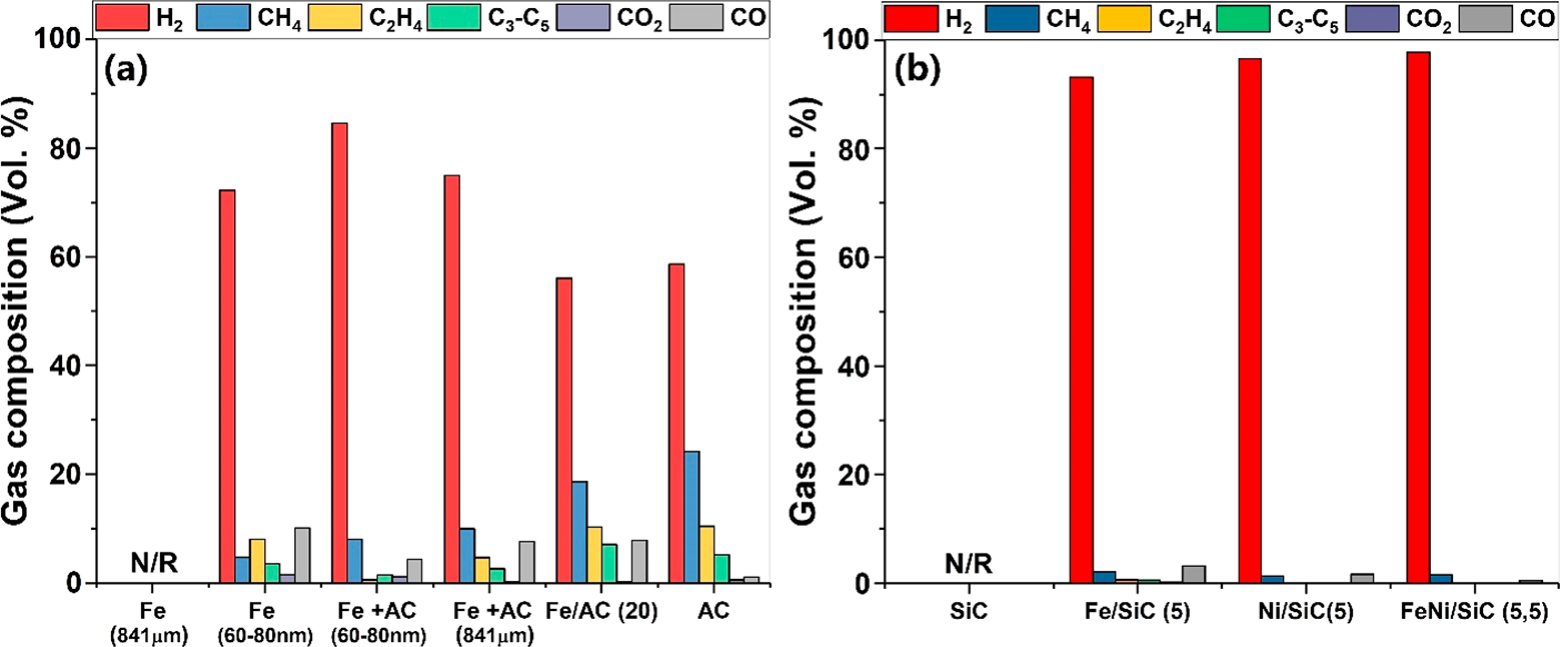
Composition of generated gases from selected catalysts for microwave-initiated dehydrogenation of hexadecane. In all cases, a 30 wt % hexadecane was mixed with catalysts before being exposed to 750 W microwave irradiation for 10 min. The generated gases were collected and analyzed by GC. “N/R” indicates no observable reaction under microwave treatment; numbers in parentheses indicate the metal loading in wt %. (b) Adapted in part with permission from ref (16). Copyright 2017 John Wiley and Sons.

In Figure 8, the catalytic performance of selected free-standing Fe particles, mechanical mixtures of Fe and AC particles, and AC- and SiC-supported Fe and Ni particles are evaluated based on the evolved gas product distribution which was analyzed quantitatively by gas chromatography (GC) (see the experimental sections for details). Hexadecane was rapidly and thoroughly dehydrogenated under microwave irradiation to yield predominantly H_2_ and solid carbon with a small amount of methane, ethylene, and C_2_–C_5_ alkanes. As a result of superfast heating under microwave irradiation, the conversion of hexadecane was calculated to be between 40 and 60% because some hexadecane reactant was rapidly volatilized at the start of the reactions,^16,17^ and the H_2_ yield obtained from the process was about 60%.^17^

Within different particle sizes, 60–80 nm Fe particles most effectively catalyzed the dehydrogenation of hexadecane under microwave irradiation, whereas importantly no measurable catalytic reaction was observed on 841 μm Fe particles. For chemical catalysis to occur, catalyst particles must reach the necessary temperature to initiate the reactions, but 841 μm Fe particles were heated poorly under microwave due to their large particle size (see Section 3.1).

However, in the mechanical mixture samples of Fe and AC, both these same-sized Fe particles mixed with AC presented a good catalytic performance under microwaves for hydrogen production. The H_2_ selectivity of two mixture samples of Fe (60–80 nm) + AC and Fe (841 μm) + AC were about 85 and 75 vol %, respectively, whereas only 72 and 57 vol % H_2_ selectivity was obtained on pure 60–80 nm Fe and AC particles (Figure 8a). Clearly, Fe particles physically and intimately mixed with AC powder improve its catalytic performance under microwave initiation. This is ascribed to the heat compensation from the AC, and possible synergistic effects occurring at their interface by interfacial polarization^18,27^ and nonequilibrium plasma^33^ as noted in the Section 3.1.2.

AC is acknowledged as an efficient microwave susceptor that has been widely used as an additive for non-microwave absorbing materials in microwave catalysis.^10,11,38−40^ AC can efficiently and rapidly convert electromagnetic energy to heat in a microwave-induced electric field. In the mixture with Fe particles, heat generated from AC could partially supply the Fe particles, enabling them to reach the necessary temperature for catalysis to occur. We note also that AC itself is catalytically active for the microwave-initiated hexadecane dehydrogenation;^11^ the hexadecane undergoes rapid decomposition to hydrogen, small alkanes (methane, ethylene, and propylene, etc.), and solid carbon. For AC, 57 vol % H_2_, 24 vol % CH_4_, 10 vol % C_2_H_4_, and 5 vol % C_3_–C_5_ were detected in the produced gases (Figure 8a). Thus, the mixture of Fe and AC combines the advantages of each constituent material under microwave irradiation; the better energy conversion of AC compensates for the poor heat production at the Fe particles by supplying them with excess heat, and the Fe metal particles give better catalytic selectivity for C–H bond activation/cleavage. This enhances the overall catalytic performance of the Fe and AC mixture for hydrogen production. Interestingly, the AC-supported 20 wt % Fe catalyst showed similar catalytic results to pure AC; it had no improvement in H_2_ selectivity. We believe the structure of Fe/AC (20) was what caused the limited improvement in H_2_ selectivity when Fe was added. In the prepared Fe/AC catalyst (Figure 7), the Fe is embedded/anchored in the pores of AC. In the earlier temperature profile of Fe/AC particles (Figure 6a), it can be seen that a similar temperature was reached by both Fe/AC (20) and pure AC. This suggests that the constituent AC in the Fe/AC catalyst was dominant in the overall response to the incoming microwaves; moreover, the limited proximity of hexadecane and the Fe particles which are embedded in AC could reduce the Fe particles’ effectiveness.

In contrast, the use of SiC as a supporting material significantly improves the catalytic performance for hydrogen production. In fact, some 93–98 vol % H_2_ was selectively produced on SiC-supported Fe, Ni, and their alloy particles (Figure 8b). In these SiC-supported metal catalysts, the SiC has a grain particle size of approximately 20–40 μm, while the loaded Fe, Ni, and Fe–Ni alloy particles are about 101, 104, and 61 nm, respectively (Figure S1).^5,16^ The Fe–Ni alloy catalyst has a smaller particle size than that of Fe and Ni nanoparticles, which also show an extremely high hydrogen selectivity. We found that the presence of nickel in an iron-based catalyst improves the dispersion of metal particles on the SiC surface and suppresses the formation of CO_*x*_.^16^

In the SiC-supported iron-based metal catalyst system, both SiC and small iron nanoparticles are excellent microwave absorbers. In addition, Fe nanoparticles are uniformly dispersed on the SiC surface and are exposed to the incoming reactant hydrocarbon molecules. Importantly, this combines the advantages of both materials that respond to microwaves. The constituent SiC is mechanically very strong and has an exceptionally high thermal conductivity. These intrinsic properties of SiC clearly contribute to the development of a thermal balance in the reaction system.^16^ Moreover, the smooth surface of SiC should enhance the molecular diffusion and improve the transport of both incoming reactants and departing products,^5^ especially when there is a possible temperature gradient built between the Fe metal particles and the SiC support. Fe nanoparticles, on the other hand, are dominant in the catalytic scission of chemical C–H bonds in the hydrocarbons.

**Figure 9:**
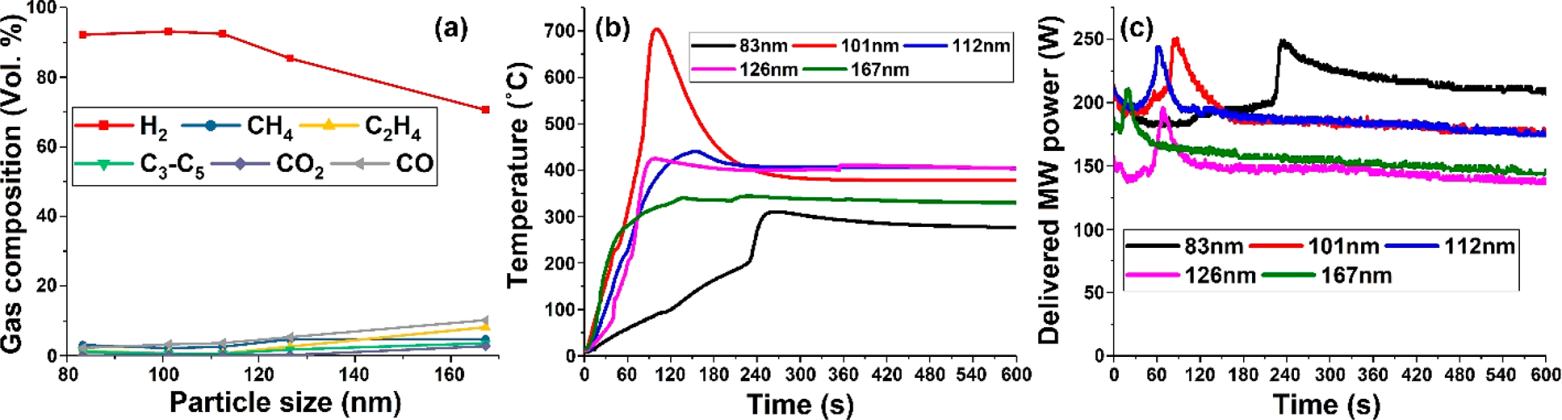
(a) Gas composition of Fe/SiC catalysts at different Fe loadings as a function of Fe particle size for the microwave-initiated dehydrogenation of hexadecane. (b) Corresponding temperature profiles of Fe/SiC catalysts and (c) delivered MW power on different Fe particle sizes.

In our earlier work^17^ on the effect of Fe loading on SiC, we concluded that the Fe loading at the lower levels, less than 10 wt %, is preferable for higher levels of hydrogen production in the evolved gases. Further investigation of the Fe particle size used in the Fe/SiC catalysts revealed that the catalytic performance is size dependent (Figure 9a). Using 10 wt % Fe loading on SiC, the size of the Fe nanoparticle is approximately 112 nm. The prepared Fe/SiC catalyst with Fe nanoparticles below 112 nm gave over 90 vol % H_2_ selectivity, whereas the nanoparticle sizes above 120 nm exhibited a decrease in hydrogen production to 85 and 72 vol % for the 126 and 167 nm sized Fe nanoparticles, respectively.

Moreover—and importantly—Fe/SiC catalysts with smaller Fe nanoparticles absorbed more microwave energy and had higher reaction temperatures (Figure 9b and c). However, the sample with the smallest Fe nanoparticles, 83 nm, showed a comparably slow heating stage for 4 min before a rapid increase in both the delivered MW power and reaction temperature. This is ascribed to the very low loading of Fe (2 wt %) which needed more time for the catalyst and substrate to reach the necessary temperature to initiate the reactions. We also note that below the Fe nanoparticle size of approximately 120 nm, which is 2–3 times the magnitude of the skin depth of iron (41.5 nm), Fe/SiC catalyst particles absorbed a large amount of microwave energy and achieved very high temperatures of over 700 °C (Figure 6b). This combination of Fe/SiC catalyst is considered the optimum for the microwave-initiated dehydrogenation of hexadecane.

## 4 Conclusion

In this work, we have demonstrated that the optimal particle size of Fe metal catalysts for the microwave-initiated deep dehydrogenation, or hydrogen-stripping from a model hydrocarbon, hexadecane, is between 80 and 120 nm. The process is clearly highly dependent on the ratio of mean particle radius to the skin depth of iron (*r*/δ) at the operating frequency. The combination of physically mixed Fe metal particles and AC has synergistic effects in improving the heating ability and subsequently enhances the overall catalytic activity of the mixture. The suitable metal and support combination of SiC-supported Fe and Ni catalysts gave exceptional yields of hydrogen production under microwave initiation. These combinations form an effective microwave-harvesting catalyst system for microwave-initiated deep dehydrogenation reactions. From this study, we hope to have begun the process of identifying the fundamental mechanism of interaction between microwave radiation and an active catalyst, which is critical for the complete catalytic process. Perhaps the most important finding from our study is the size-induced transition of particulate catalytic metals from a microwave “reflector” to “absorber” and that electronic transition must be carefully considered in the selection of efficient metal catalysts. Thus, the physical size of small conducting metal catalyst particles will decisively affect their heating characteristics and hence their catalytic performance under microwave initiation.

Since the heating mechanism of microwaves is fundamentally different from conventional thermal heating, incoming microwaves will interact directly with each material independently. Thus, the catalyst design for microwave-initiated catalysis should carefully consider the microstructural, electrical, and magnetic parameters, such as particle size, electric conductivity, saturation magnetization, and dielectric and catalytic properties of all constituent materials.

Finally, in this application of microwave-initiated catalysis, we note that this work provides an excellent route for producing ultralow CO_2_—or even CO_2_-free—hydrogen within a carefully constructed catalyst system design/optimization. The guidance outlined here on catalyst design and optimization through size-dependent effects will hopefully be of help in other important catalytic chemical reactions initiated by microwaves, particularly those in producing hydrogen from fossil fuels themselves without CO_2_ emission.

## Supporting Information Available

Additional details of the XRD characterization of SiC-supported Fe, Ni, and Fe–Ni alloy catalysts (Figure S1), SiC raw materials (Figure S2), and representative mixture samples of physically mixed 60–80 nm Fe metal particles and activated carbon (Figure S3) (PDF)

## Notes

The research materials supporting this publication are contained within the paper and its associated Supporting Information. All other relevant data is available from the corresponding author upon reasonable request.
