# Supplementary material for: Size-Dependent Microwave Heating and Catalytic Activity of Fine Iron Particles in the Deep Dehydrogenation of Hexadecane
Source: Chem Mater. 2022 May 13;34(10):4682–93. doi: 10.1021/acs.chemmater.2c00630 (PMC9134345; doi:10.1021/acs.chemmater.2c00630)
Supplement: Supplementary file 1 — cm2c00630_si_001.pdf [file cm2c00630_si_001.pdf]

Supplementary Information for

Size-Dependent Microwave Heating and Catalytic Activity of Fine Iron Particles in the Deep Dehydrogenation of Hexadecane

Xiangyu Jie[1*], Roujia Chen[1], Tara Biddle[1], Daniel R. Slocombe[2*], Jonathan Robin Dilworth [1], Tiancun Xiao[1] and Peter P. Edwards[1*]

1. Inorganic Chemistry Laboratory, Department of Chemistry, University of Oxford, South Parks Road, Oxford OX1 3QR, UK.
2. School of Engineering, Cardiff University, Queen's Buildings, The Parade, Cardiff, CF24 3AA, UK.

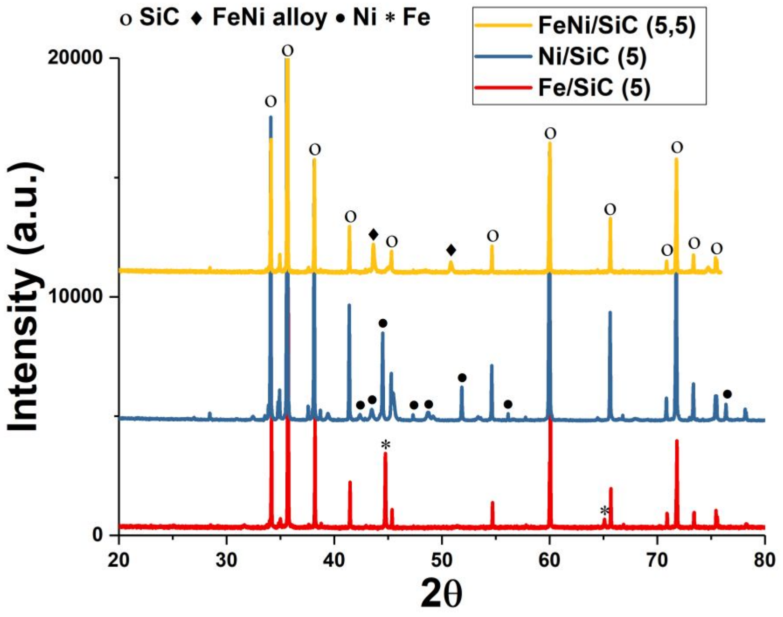

Figure S1. XRD patterns of SiC supported Fe, Ni and FeNi alloy catalysts. The crystallite size of the Fe, Ni and FeNi alloy nanoparticles calculated based on the Scherrer equation are 101, 104 and 61 nm, respectively.

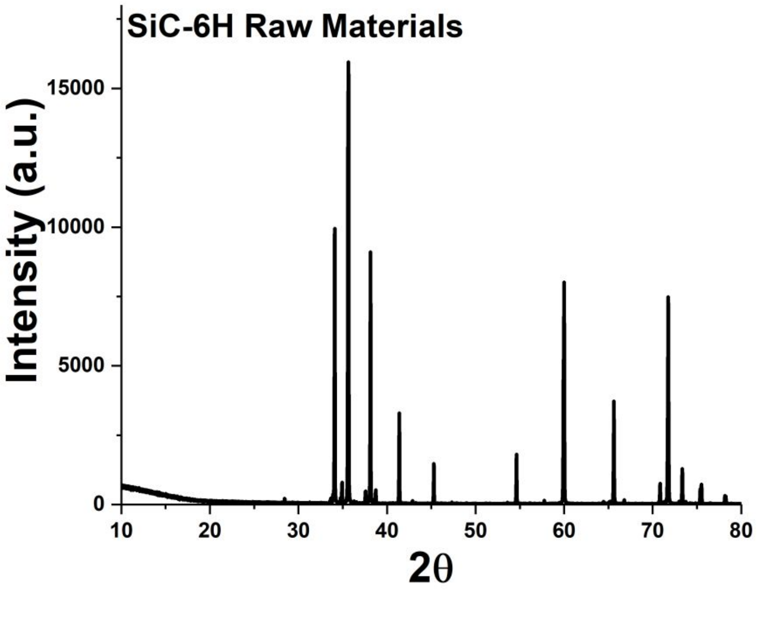

Figure S2. XRD patterns of SiC-6H Raw Materials.

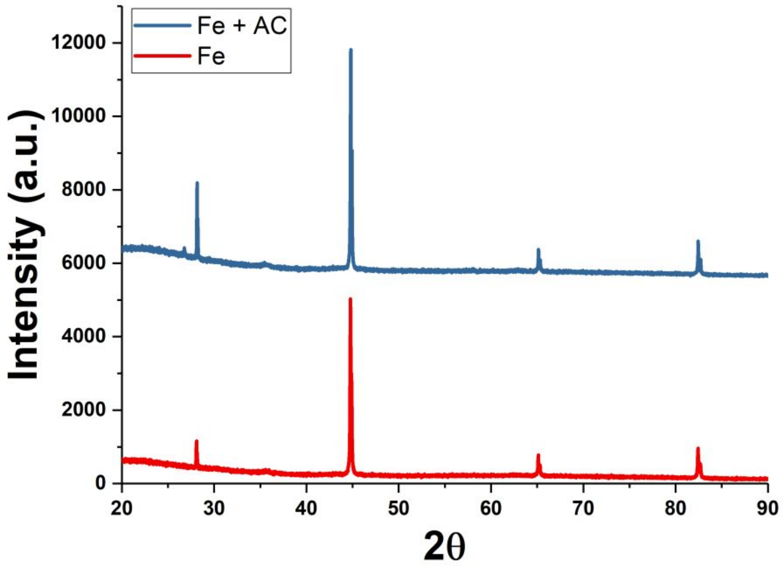

Figure S3. Representative XRD pattern of physically mixed 60-80nm Fe metal particles and activated carbons (AC).
